# A Comprehensive Analysis of the Abdominal Aortic Aneurysm Growth Rate in the Spanish Population

**DOI:** 10.3390/jcm14134720

**Published:** 2025-07-03

**Authors:** Olga Peypoch, Laura Calsina Juscafresa, Antón Vega-Méndez, Bárbara Lobato-Delgado, Joan Fité, Begoña Soto, Lluis Nieto, Mireia de la Rosa Estadella, Ager Uribezubia, Jose-María Romero, Emma Plana, Manuel Miralles, Albert Clarà, Jaume Dilmé, José Manuel Soria, Mercedes Camacho, Angel Martinez-Perez, Maria Sabater-Lleal

**Affiliations:** 1 Department of Vascular and Endovascular Surgery, Hospital de la Santa Creu i Sant Pau, 08025 Barcelona, Spain; opeypoch@santpau.cat (O.P.); jfite@santpau.cat (J.F.); bsoto@santpau.cat (B.S.); jromeroc@santpau.cat (J.-M.R.); jdilme@santpau.cat (J.D.); 2 Unit of Genomics of Complex Disease, Institut de Recerca Sant Pau, IR SANT PAU, 08041 Barcelona, Spain; antonvegamendez@gmail.com (A.V.-M.); blobato@santpau.cat (B.L.-D.); mrosae@santpau.cat (M.d.l.R.E.); auribezubia@santpau.cat (A.U.); jsoria@santpau.cat (J.M.S.); mcamacho@santpau.cat (M.C.); amartinezp@santpau.cat (A.M.-P.); 3 Department de Cirurgia i Ciències Morfològiques, Universitat Autonoma de Barcelona (UAB), 08193 Barcelona, Spain; 4 Vascular Surgery Department, Hospital del Mar de Barcelona, 08003 Barcelona, Spain; lcalsina@hmar.cat (L.C.J.); aclara@hmar.cat (A.C.); 5 Department of Medicine and Health Sciences, Universitat Pompeu Fabra, 08003 Barcelona, Spain; 6 Grupo Acreditado de Hemostasia, Trombosis, Arteriosclerosis y Biología Vascular, Instituto de Investigación Sanitaria La Fe, 46026 Valencia, Spain; emma_plana@iislafe.es (E.P.); mirallesm@telefonica.net (M.M.); 7 Servicio de Angiología y Cirugía Vascular Hospital Universitario y Politécnico La Fe, 46026 Valencia, Spain; 8 Facultad de Medicina, Universitat de València, 46010 Valencia, Spain; 9 Centro de Investigación Biomédica en Red de Enfermedades Cardiovasculares (CIBERECV), 28029 Madrid, Spain; 10 Centro de Investigación Biomédica en Red de Enfermedades Raras (CIBERER), 28209 Madrid, Spain; 11 Cardiology Unit, Department of Medicine, Karolinska Institutet, Center for Molecular Medicine, 171 76 Stockholm, Sweden

**Keywords:** Abdominal Aortic Aneurysm (AAA), risk prediction models, AAA screening, risk factors

## Abstract

**Objective:** The risk of Abdominal Aortic Aneurysm (AAA) rupture is associated with the aneurysm size and growth rate. This study aims to provide a global description of growth rates per intervals of AAA diameter size for individuals in the Spanish population, to understand possible comorbidities associated with growth rate variability, and to assess practitioners on safe follow-up visits for AAA patients. **Methods:** We present the Triple-A Barcelona Study (TABS), a new hospital-based longitudinal study recruiting consecutive individuals with AAAs in Barcelona. So far, 469 individuals with measurements of the abdominal aortic diameter, along with anthropometric, clinical information, and blood samples for most follow-up visits, have been recruited. Statistical modeling was performed to identify the most relevant predictors of the diameter size and expansion in individuals with AAAs using linear mixed-effect models. **Results:** The average growth rate per interval was 0.78 (2.34) mm/year for aneurysms with an initial diameter between 30 and 40 mm, 1.22 (3.34) mm/year for aneurysms with an initial diameter between 40 and 50 mm, and 4.12 (5.09) mm/year for aneurysms with an initial diameter equal to or greater than 50 mm. The main factors determining the growth rate beyond the aortic diameter are sex and related comorbidities (COPD and DM). The estimated time to reach the surgical threshold for individuals with small aneurysms exceeded 10 years, on average. **Conclusions:** Overall, this study serves as a promising step towards the development of better prediction tools to assess clinical decisions in AAA patients in the Spanish population and to guide future screening policies.

## 1. Introduction

Abdominal Aortic Aneurysms (AAAs) are local dilatations of the abdominal aorta characterized by the structural deterioration of the vascular wall leading to progressive dilatation and, potentially, rupture of the abdominal aorta [1]. The risk of rupture is associated with the aneurysm size and growth rate [2,3,4,5,6,7,8,9,10,11], and it has a high mortality rate [12], representing the 12–15th leading cause of death in persons over 55 years of age in the USA and Europe.

Surgery is the only treatment option to prevent AAA-related death (AAA rupture). The threshold for surgical treatment is defined at a 55mm maximum transverse diameter in men, according to the European Society for Vascular Surgery (ESVS) guide, or in cases of rapid diameter growth, defined as 5 mm in 6 months or 10 mm in 12 months [13].

Screening is recommended in some countries for men over 65, or for men over 65 that have ever smoked [14], but while the current literature indicate that there is an important reduction in mortality due to AAAs when screening is performed, there is currently no population screening program in Spain, and there is no clear agreement on whether the implementation of such screening would be viable and economically feasible. Small local screening studies have been performed, but not systematically. While these studies suggest that AAA deaths and the number of emergent rupture surgeries would clearly be reduced, they all point to the need for improved evidence based on local prevalence and local economic estimates, before population-based screening programs can be considered [15,16,17].

The availability of studies on AAA expansion are sparse. Here, we present the Triple-A Barcelona Study (TABS), the first large Spanish prospective hospital-based longitudinal study enrolling consecutive individuals with AAAs treated in two public hospitals in Barcelona, which aims to provide a global description of growth rates per intervals of AAA diameter size, to pinpoint possible comorbidities associated with the growth rate in individuals in the Spanish population, and to assess optimal surveillance intervals for practitioners.

## 2. Materials and Methods

### 2.1. Sample Inclusion

TABS is a prospective longitudinal registry started in 2019. All individuals identified incidentally with AAAs, defined as those with dilation in the infrarenal abdominal aorta with a diameter greater than 30 mm [13] that visited the Vascular and Endovascular Surgery Units of Hospital de la Santa Creu i Sant Pau and Hospital del Mar (Barcelona, Spain) from 2019 to March 2025 were invited to participate in the project. Patients who did not consent to participation were not considered for enrollment. Upon enrollment, participants’ medical histories were carefully reviewed, and all previous measures of aortic diameter and available clinical information was included retrospectively in the database. All participants had at least one measurement of the abdominal aortic diameter, either by computed tomography scan (CT scan) or by ultrasound along with anthropometric and clinical information, and blood samples for most follow-up visits. The first measurement was considered the baseline. All measurements following the baseline measurement were used until aortic repair or rupture, or death. Individuals that were excluded from surgery after they exceeded the surgical threshold (due to concomitant pathology, AAA anatomy, or patient rejection of surgery) were also considered for inclusion, and their retrospective data, if existing, were recorded. All patients were asked to give informed consent to participate in this study, and to be included in a clinical database. Type B dissections that progressed to AAAs, saccular aneurysms, mycotic aneurysms, visceral aneurysms, or thoracic aneurysms were excluded. Aortic diameter was assessed using either ultrasound or CT scan images. Additional information about data collected from all participants is detailed in Supplementary Materials. We followed CONSORT guidelines for appropriate reporting. The study protocol conforms to the ethical guidelines of the 1975 Declaration of Helsinki and all procedures have been approved by the ethical committee of Hospital Sant Pau (IIBSP-OMI-2019-102, Ref. 20/015, approved on 15 January 2020). The protocol has been registered on ClinicalTrials.gov with identification number NCT06827652.

### 2.2. Data Cleaning

Both ultrasound and CT scan measurements were included in the analyses. To deal with variability in diameter measurements between ultrasound and CT scans, we adjusted the ultrasound diameters to align with the CT scan measurement using an estimation calculated within the cohort, which resulted in the addition of 2.21 mm to the ultrasound measurements. Ultrasound exams were performed by the same technician at each hospital and CT images were validated by both a radiologist and vascular surgeon, minimizing uncontrolled variability. The rationale to calculate the adjustment factor is described in more detail in Supplementary Materials. Missing values for comorbidities, weight, height, and smoking were estimated by applying the same value as in the previous visit if the value had been recorded before. Visit entries with missing values for aortic diameter were excluded.

### 2.3. Statistical Modeling and Selection of Main Factors Associated with Aortic Diameter

Statistical modeling of aortic diameter defined in mm was performed to identify the most relevant predictors of diameter size in individuals with AAAs. We selected AAA risk factors based on the literature, including sex, age, smoking status (ever-smokers, non-smokers), dyslipidemia, presence or history of diabetes mellitus (DM), chronic obstructive pulmonary disease (COPD), cerebrovascular events (CVEs), other cardiovascular (CV) events, peripheral artery disease (PAD), kidney disease, or presence of other aneurysms. We used linear mixed models using random effects to adjust for the individual effects. The response variable was the aortic diameter, with the risk factors, years from study entry, the aortic diameter at the initial visit (baseline), and the interaction between years from study entry and the aortic diameter at the initial visit as covariates. The 95% confidence intervals (CIs) and *p*-values were computed using a Wald t-distribution approximation. All analyses were performed in R v.4.5.1 [18,19]. Further details for the statistical analyses are reported in the Supplementary Materials.

### 2.4. Statistical Modeling and Selection of Main Factors Associated with Aortic Diameter Growth

For the 291 patients that had at least 2 measures of aortic diameter, we estimated 2 measures of growth rate: (1) “*average growth rate*” was calculated for each individual as the beta coefficient of time elapsed from the first visit (in years), derived from the linear regression aortic diameter–time; (2) “*total growth rate*” was calculated as the difference between the first and last measurements, divided by the time elapsed in years between the two measurements. All measures were expressed in mm per year. The 2 growth rates were calculated globally and per each of the 3 intervals of baseline aortic diameter: 30–39.9 mm, 40–49.9 mm, and ≥50 mm. Risk factors based on the literature, including sex; age; height; body mass index (BMI); smoking status (ever-smokers, non-smokers); hypertension; dyslipidemia; presence or history of DM, PAD, CV, CVE, COPD, or kidney disease; and presence of other aneurysms, were explored for their effect on average or total AAA growth rate. All models additionally included age at basal measure and diameter at baseline as covariates. Finally, we included a random effect to account for the underlying effect of the variation in diameter between and within individuals. The model’s total explanatory power was evaluated with R2 statistic. All *p*-values and 95% CIs were computed using a Walt t-distribution approximation. Because not all growth patterns seem to show a linear trend, we also explored additional models that considered the non-linearity of aneurysm growth (see Supplementary Materials).

## 3. Results

### 3.1. Cohort Description

A total of 471 individuals, 454 males and 17 females (3.6%), with AAA were included. In the study period, 2184 follow-up visits with imaging data were recorded, of which 430 were excluded. In total, 294 visits were excluded for being performed after surgical repair of the aneurysm, 43 visits because they documented an aortic diameter < 30 mm, 20 visits because they lacked specification of the imaging technique, and 73 visits were excluded as they were annotations indicating cessation of patient follow-up, leaving a total of 1754 suitable observations (1202 ultrasounds, 552 CT scans). There was an average of 3.7 visits per patient (SD = 3.5), with a maximum of 20 visits for a single individual, and the average follow-up time was 2.5 years (SD = 2.9), with the longest follow-up time extending to 15.2 years. Only 17 women were available, with an average of 3.5 images per woman (33 ultrasound and 26 CT scans). Among all patients, the mean age was 73.3 years (SD = 7.75). The mean (SD) aortic diameter at study entry was 49.75 (14) mm. Information about lifestyle habits and other diseases is summarized in Table 1.

### 3.2. Growth Rate by Aortic Diameter Intervals

**Figure 1:**
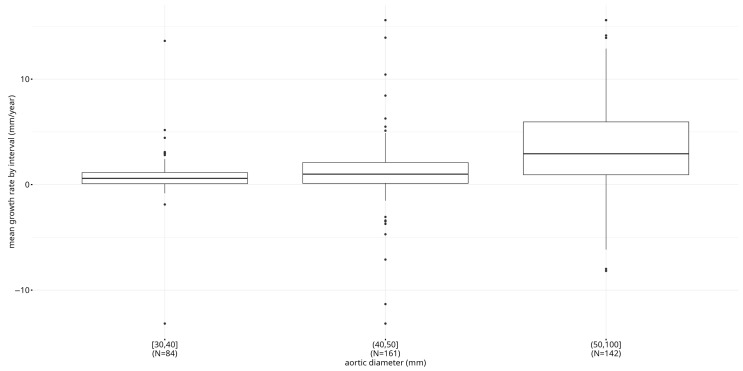
Boxplot of aortic diameter mean growth rate (mm/year) by interval in the TABS sample. Boxes show the interquartile range and the median. Whiskers extend from the smallest and the largest values within 1.5 the interquartile range.

Our results showed a significant increase in growth rates for increasing diameter at baseline intervals: the average (SD) growth rate, as calculated per linear regression, was 0.78 (2.34) mm/year for aneurysms with an initial diameter between 30 and 40 mm, 1.22 (3.34) mm/year for aneurysms with an initial diameter between 40 and 50 mm, and 4.12 (5.09) mm/year for aneurysms with an initial diameter equal to or greater than 50 mm. The total growth rate, as calculated by dividing the total difference in the aortic diameter by the time elapsed was 0.79 (2.48) mm/year for the aneurysms with an initial diameter between 30 and 40 mm, 1.27 (3.55) mm/year for aneurysms with an initial diameter between 40 and 50 mm, and 4.37 (5.46) mm/year for aneurysms with an initial diameter equal to or greater than 50 mm. There was substantial variation between the individuals with comparable diameters at baseline using both measures, suggesting that additional factors contribute to the variability in growth rate (Figure 1). The women tended to show higher growth rates, although, due to the limited number of women in our sample, we could not calculate reliable growth rates specific for women.

### 3.3. Selection of Best Variables Influencing AAA Diameter

We first calculated a reference model to explore the main variables affecting the AAA diameter including the age at inclusion, sex, and smoking status, to identify factors influencing the aortic diameter in AAA patients.

We then assessed whether adding additional information on related diseases increased the efficiency of the model and included DM, dyslipidemia, PAD, CVE, CV, COPD, kidney disease, and other aneurysms to the previous model. Apart from the random effects accounting for the baseline diameter and the years since first visit, the best and most parsimonious model for predicting aortic diameter included dyslipidemia (beta = 0.39, CI95% [0.04, 0.74], *p*-value = 0.03). The effect of sex was not significant. The full model’s total explanatory power was R2 = 0.97. The marginal R2, related to fixed effects alone, was 0.62.

**Figure 2:**
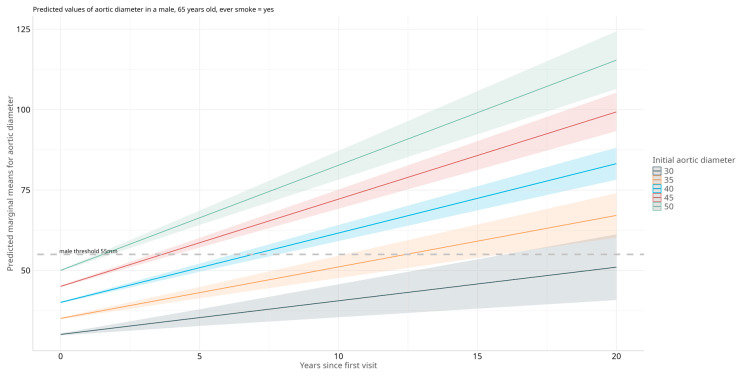
Predicted aortic diameter over time calculated for a 65-year-old male individual with dyslipidemia. The Y axis represents the predicted marginal means for aortic diameter measured in mm. The X axis represents the years since the first medical visit. The colors represent five hypothetical aortic diameters at the first visit, and the shaded areas indicate the 95% confidence intervals for predictions. The dashed gray line represents the clinical threshold for intervention (55 mm for males), included in the visualization to facilitate comparison with clinical standards.

Based on this model, to visualize the predicted growth trajectories for every hypothetical case with certain covariates, we fixed specific variables representing a male individual with aortic diameters at first visit of 30 mm, 40 mm, 45 mm, and 50 mm (Figure 2) as an example of what could be used to guide clinical decisions. CIs (95%) were calculated and plotted to illustrate the uncertainty around the predicted values. In this example, given a 65-year-old male with dyslipidemia presenting an aortic diameter of 40mm, the model would predict that, on average, it would take 6.85 years (6.1–7.7 CI at 95%) for the aortic diameter to reach 55 mm.

### 3.4. Selection of Best Variables Influencing AAA Growth Rate

To obtain average predictors for all individuals in specific diameter intervals that could help anticipate global estimates of time to reach a surgical diameter per interval, we aimed to model the growth rate. For this, we tested the association of a basic model including age, ever-smoker status, baseline diameter, and sex to predict the two computed growth rate measures defined before. When we used the average growth rate in mm/year as an outcome, we observed that the model explained a small proportion of the variance and the only significant parameter explaining the average growth rate was the baseline aortic diameter; the growth rate was bigger at larger aortic diameters (*p*-value = 1.7 × 10^−6^). A full model including additional clinical variables found female sex (beta = 2.31, CI95% [0.21, 4.41], *p*-value = 0.03) and the presence of COPD (beta = 1.11, CI95% [0.05, 2.17], *p*-value = 0.04) to be associated with a higher average growth in addition to the aortic diameter at baseline. Using a full model to analyze the total AAA growth rate, we identified suggestive effects for the association of female sex (beta = 2.35, CI95% [0.04, 4.66], *p*-value = 0.05) and COPD (beta = 1.00, CI95% [−0.11, 2.12], *p*-value = 0.08) and a suggestive protective effect of DM (beta = −1.10, CI95% [−2.21, 0.02], *p*-value = 0.05) in addition to the effect of the aortic diameter at baseline.

Finally, we calculated the same models using the growth trend as an outcome, to better explain the AAA non-linear growth behavior of the aneurysms, and we did not see a relevant improvement in the models when compared to previous analyses with average growth or total growth.

### 3.5. Models to Predict Estimated Time to Surgery

With the aim to use our data to assess surveillance intervals, we then used the *average growth rate*, calculated given specific diameter intervals, to infer the expected time to reach an aortic diameter of 55 mm for males and 50 mm for females, as a measure that could help anticipate the time to surgery based on average growth rates in our sample for all individuals with AAA diameters at a certain interval. Finally, given these values, we calculated an estimated optimal time to next visit in years, allowing for a maximum of 1% or 5% of the patients to exceed the surgery limit during this time interval, stratifying by sex.

**Figure 3:**
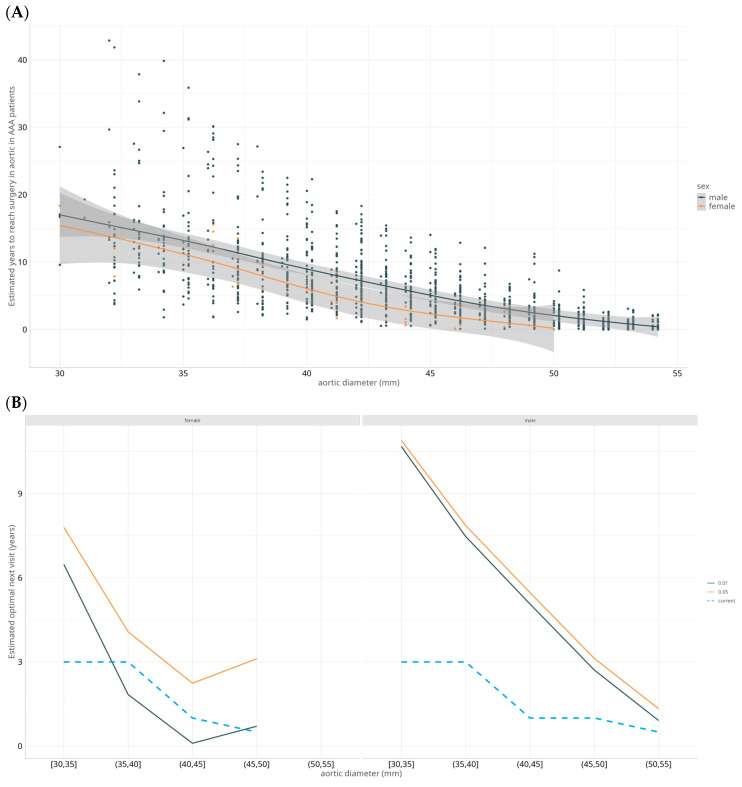
Estimated time to surgery threshold: (**A**) Estimated time in years to reach the threshold for surgery (55mm in men and 50 mm in women) by aortic diameter. Shaded areas indicate the 95% confidence intervals for predictions. (**B**) Estimated optimal next-visit interval for females (left) and males (right) considering that no more than 1% (black) or 5% (orange) of the individuals will pass the threshold for surgery before that visit. Dashed-blue line indicates the visits recommended by current guidelines.

The estimated time to reach an aneurysm size of 55 mm (threshold for surgery) in individuals with small aneurysms (between 30 and 40 mm) exceeded the currently used surveillance intervals in Spain (Table 2). For example, for male individuals with an initial aortic diameter between 35 and 40 mm, the time to reach 55 mm would be over 10 years on average (Figure 3), and in 7.5 years, only 1% of the males would reach 55 mm (Table 2). However, in individuals with aortic diameters between 50 and 55 mm the number of visits and intervals between visits (between 3 and 6 months) seem to match the calculated time better when 1% of the males would reach 55 mm.

Based on the data and predictions in the present work, we have included, in the Supplementary Materials, a predicted number of visits that could be redirected to screening a high-risk population (male smokers between 60 and 65) and a speculative number of additional AAA cases that could be detected considering AAA incidence in this group.

## 4. Discussion

The optimization of screening intervals for AAA expansion may depend on specific populations and, while European guidelines exist, how often surveillance should be offered to patients with small aneurysms is still subject to debate, and studies in different centers give different results [20,21,22,23,24,25]. Moreover, understanding additional risk factors that may help reduce the expansion of small aneurysms is still a crucial medical need. Here, we present TABS, a longitudinal cohort study aiming at exploring additional clinical risk factors determining AAA expansion and improving screening intervals enrolled in surveillance programs in Spain.

### 4.1. Clinical Determinants of Aortic Diameter in Individuals with AAA

Our results detected a significant effect of dyslipidemia on the aortic diameter in AAA patients, which agrees with current knowledge from genetic analysis results highlighting the critical role of lipids and lipid metabolism in AAA pathogenesis [26]. Our results could not demonstrate clear relevant effects of additional clinical factors that have been reported before to determine the aortic diameter beyond age, sex, smoking, and the initial aortic diameter, like hypertension or related diseases. Given previous evidence in larger studies [27], we hypothesize that these factors with a smaller effect on the aortic diameter could not be detected with our current sample size. Additionally, since our cohort is a hospital-based observational study, most of our study subjects are undergoing statin, anti-hypertension, and lipid-lowering medication treatment, which complicates the assessment of the actual impact of these comorbidities on AAA growth. For example, a previous study of aneurysm growth in 1743 UK patients with AAAs [28] found an association between diabetes and smoking with diameter growth but not dyslipidemia. Others have evaluated risk scores based on clinical data to improve the identification of people at risk of AAAs. In an analysis of 1570 cases of AAAs from UK Biobank [29], the generation of a risk score based on the patient’s age, height, weight, blood pressure, baseline cardiovascular disease, diabetes, and antihypertensive and cholesterol-lowering medication use, was proven to be significantly more effective at improving the prediction of people at risk compared to a basal model including the patient’s age, sex, smoking status, and family history. Future studies are needed to clarify the usefulness of adding additional clinical information from patients with a diagnosed AAA for a more personalized estimation of the aortic diameter.

### 4.2. AAA Expansion Rates per Diameter Interval and Clinical Determinants of Expansion

The average rate of expansion increased by the aortic diameter interval and ranged between approximately 0.69 mm/year in smaller diameters and 4.07 mm/year in diameters over 55 mm. Interestingly, our findings suggest different significant parameters explaining the aortic diameter and average growth rate. For the growth rate, the main determinants were clearly the aortic diameter interval and sex, which is in line with previous results and agrees with surveillance guidelines [28], but we could also see suggestive effects of the presence of COPD as a risk factor, and DM as a protective factor for higher growth rates, which suggests that considering comorbidities could predict surveillance times and expected risk of rupture better for AAA patients. We did not find an association with smoking, although this could be explained by the limited number of never-smokers in our population, and by the bias caused by individuals with larger diameters being recommended for smoking cessation.

Despite adding information about clinical data and comorbidities, our data show that the growth patterns of AAAs vary significantly among AAA patients, with some individuals exhibiting small gradual increases in diameter over time, while others experience rapid dilatation. The identification of measurable factors that can aid in predicting the propensity for rapid growth (and potential rupture) of AAAs represents one of the main challenges in AAA clinical guidelines. These will probably include genetic markers, as well as full proteomic screens and biomechanical or morphofunctional imaging data to identify novel factors that can help move towards a more personalized assessment of aortic diameter growth and surveillance intervals. In this direction, the TABS cohort, with available DNA and RNA data, along with plasma samples collected in longitudinal patient visits, constitutes a powerful resource to move towards a promising improvement in growth prediction, becoming one step closer to precision medicine.

We have demonstrated here that adding additional information beyond aortic diameter and sex can help anticipate the time to surgery. However, additional factors may affect the rupture risk and should be considered before any changes are implemented, for example, differences in the diameter when rupture occurs, which may vary largely between individuals, or differences in the compensatory mechanisms of the aorta to disease progression. In this regard, larger cohorts with big data collections, including different layers of longitudinal omics data, will be essential to provide better prediction models for clinical use.

### 4.3. Optimization of Surveillance Intervals

With the aim of exploring optimal surveillance intervals in the Spanish population, we calculated the expected time to reach a surgical aortic diameter based on the average growth rates in the population and estimated an optimal time to next visit in years, allowing for a maximum of 1% or 5% of the patients to exceed the surgery limit during this time interval. While we did not have enough statistical power to safely establish optimal surveillance times in women, the results suggest that the current surveillance times used in Spain seem overly conservative for small aneurysms in men. Specifically, we observed that it would take male individuals with an initial aortic diameter between 35 and 40 mm more than 7 years to reach a surgical diameter and therefore, surveillance intervals could be safely increased, with significant cost savings, while males with diameters over 50 mm could benefit from even shorter intervals than currently used for safer surveillance. This agrees with previous reports in the UK [28] and elsewhere [30,31,32,33,34], indicating that individuals at lower diameters could safely be screened every 36 months while screening at larger diameters is recommended with shorter intervals.

The cost–utility long-term results from the screening programs for 65- to 75-year-old men have shown that population screening programs reduce mortality and are cost-efficient. However, the low prevalence of AAAs in Spain indicates that cost-efficiency would only be reached in population subgroups of individuals at risk [35,36,37,38]. Given that our data suggest that increasing surveillance intervals at lower diameters would be safe, further work would be advisable to elucidate if these extra visits could be invested in to screen the population at risk in Spain (male smokers between 60 and 65) [13,35,39,40,41]. Towards this aim, based on the data and predictions in the present work and assuming a predicted average number of extra unnecessary visits per year estimated for the total Spanish population, our estimation would suggest that performing these extra unnecessary visits on male smokers between 60 and 65 years old would help prematurely identify 521 novel AAAs/year in Spain at no extra cost, considering a prevalence of AAAs in male smokers of 2.67% [17]. While specific numbers are speculative, further cost-effectiveness analyses considering larger regions in Spain would be advisable to reconsider surveillance intervals and screening protocols.

### 4.4. Strengths and Limitations

We present a new cohort of individuals with AAA and follow-up data on clinical information and biological samples taken at multiple follow-up visits. This represents a valuable resource with huge potential in the study of AAA progression. However, data collection is ongoing, and we are aware of the limited existing sample size, which impacts the power of our analyses and our ability to identify significant associations with smaller effects. Additionally, several cohort characteristics might have biased the results. First, due to the lower prevalence of AAAs in women, the limited number of women in our cohort may have biased our results towards factors affecting AAA growth in male individuals, and makes our estimations of growth in females unreliable. Second, TABS is a hospital-based patient-ascertained cohort, with most patients under medication treatment, which can affect the association of several parameters. Therefore, the estimates are not population-based and collider bias may occur. Additionally, while all of the ultrasound measurements were performed by the same technician and with the same sonography machine at each hospital, there is an inherent unquantified measurement error that might affect growth rates. Moreover, because we rely on existing medical exams, our data do not include paired CT–ultrasound scans, which would have constituted the ideal scenario to validate the Bland–Altman adjustment. Third, smoking habits, which are a strong risk factor for AAA progression, are not representative of those in the general population, which might bias the results. Fourth, we are also aware that our cohort includes mainly individuals with European ancestry. Given the plausible interaction with other population-specific confounders, the extension of the predictive variables on AAA risk in other populations will need further verification. Fifth, a large proportion of our patients were detected at large diameters, reducing the power to more accurately predict aortic diameter growth at smaller baseline diameters, and increasing possible measurement error between closer measures. Sixth, although our study provides growth rate estimates that can support surveillance scheduling in clinical practice, we acknowledge that AAA progression is highly heterogeneous and that relying solely on diameter thresholds (55 mm in men and 50 mm in women) may overlook important patient-specific factors. In fact, cases of rupture below these cut-offs have been documented. While our dataset does not currently include biomechanical imaging or tissue-level data, we recognize the value of incorporating such parameters into future models to better predict expansion and rupture risk. Finally, while we considered that redirecting medical visits to screen high-risk individuals in the population would not add extra costs, the implementation of such a screening program would have an additional startup budget impact, which has been omitted for the present estimations.

## 5. Conclusions

In conclusion, this work presents a novel longitudinal study of AAA patients with repetitive clinical and biological data. Our findings provide information about growth rates in the Spanish population with aortic diameters > 30 mm followed up by vascular surgery services in Spanish hospitals. The findings help identify additional comorbidities that could affect the growth rate, and suggest that the current surveillance times used in Spain could be optimized and that unnecessary visits could be redirected to screen a subgroup of a population at risk.

We anticipate that further research efforts, including future expansion of the TABS cohort, are required to refine the best model to predict growth, which will involve incorporating high-dimensional data and larger sample sizes. Nevertheless, this study serves as a promising step towards the development of better prediction tools to assess clinical decisions in AAA patients in the Spanish population and to guide future screening policies.

## Figures and Tables

**Table 1:** Descriptive summary of the TABS cohort (N = 469) per aortic diameter interval. Of note, 2 individuals exceeded the interval upper limit with aortic diameters >100 mm and are not included in this table.

Variable	[30,40] (n = 121)	(40,50] (n = 161)	(50,100] (n = 187)	Total (n = 469)
sex [female], %, n	4.1, 5	5.6, 9	1.6, 3	3.6, 17
mean age (SD), n	69.54 (7.24), 121	72.10 (7.43), 161	73.89 (8.10), 187	72.15 (7.83), 469
mean height (SD), n	171.55 (6.97), 118	170.37 (7.10), 157	169.90 (6.92), 148	170.54 (7.01), 423
mean weight (SD), n	79.91 (10.61), 118	80.57 (13.36), 156	78.95 (12.84), 149	79.81 (12.45), 423
smoking [never], %, n	14.0, 17	8.1, 13	12.8, 24	11.5, 54
smoking [before 6 months], %, n	48.8, 59	51.9, 83	55.1, 103	52.4, 245
smoking [current smoker], %, n	37.2, 45	40.0, 64	32.1, 60	36.1, 169
hypertension [yes], %, n	71.9, 87	80.0, 128	79.0, 147	77.5, 362
dyslipidemia [yes], %, n	69.4, 84	74.4, 119	62.6, 117	68.4, 320
diabetes [yes], %, n	25.6, 31	22.5, 36	26.2, 49	24.8, 116
PAD [yes], %, n	15.7, 19	15.8, 25	9.5, 17	13.3, 61
other_aneurysms [yes], %, n	18.1, 21	14.6, 23	18.1, 32	16.9, 76
cve [yes], %, n	7.4, 9	8.8, 14	7.0, 13	7.7, 36
cvd [yes], %, n	18.2, 22	24.1, 38	36.3, 65	27.3, 125
copd [yes], %, n	16.5, 20	26.6, 42	26.8, 48	24.0, 110
kidney_disease [yes], %, n	12.4, 15	15.8, 25	17.9, 32	15.7, 72
antihypertensives [yes], %, n	66.7, 60	79.6, 82	76.7, 125	75.0, 267
aines_aspirin [yes], %, n	56.7, 51	55.3, 57	65.0, 106	60.1, 214
glucocorticoids [yes], %, n	3.3, 3	3.0, 3	3.2, 5	3.2, 11
statins [yes], %, n	75.6, 68	77.5, 79	77.3, 126	76.9, 273
antidiabetics [yes], %, n	23.3, 21	15.5, 16	20.2, 33	19.7, 70
anticoagulants [yes], %, n	7.8, 7	14.6, 15	17.8, 29	14.3, 51
surgery [no], %, n	94.1, 111	92.3, 131	23.8, 40	65.9, 282
surgery [yes(EVAR, endovascular repair)], %, n	3.4, 4	6.3, 9	55.4, 93	24.8, 106
surgery [yes(open surgery)], %, n	2.5, 3	1.4, 2	20.8, 35	9.3, 40
mean BMI (SD), n	27.18 (3.35), 117	27.71 (3.93), 156	27.28 (3.75), 148	27.41 (3.71), 421
mean aortic_diameter (SD), n	35.75 (3.11), 121	44.92 (2.99), 161	62.25 (11.39), 187	49.46 (13.35), 469
mean number_visits (SD), n	6.01 (3.91), 121	4.58 (3.32), 161	1.53 (1.42), 187	3.74 (3.47), 469
mean years_follow_up (SD), n	5.03 (3.61), 121	2.73 (2.63), 161	0.27 (0.69), 187	2.34 (3.09), 469

Values for age, height, and weight were taken for the first visit for each individual within the interval. For all diseases and medications, we counted all individuals with at least one positive value within the interval. N: number of individuals; SD: standard deviation; PAD: peripheral artery disease; CVE: cerebrovascular event; CVD: cardiovascular disease; COPD: chronic obstructive pulmonary disease; NSAIDs: non-steroidal anti-inflammatory drugs; EVAR: endovascular aneurysm repair.

**Table 2:** Optimal visit prediction.

	Male					
	Years to Next Visit at 5%	Years to Next Visit at 1%	Current Years to Next Visit	Number of Visits at 5%	Number of Visits at 1%	Current Number of Visits Until 55 mm
[30,35]	10.9	10.7	3.0	4.5	5.2	15.6
(35,40]	7.9	7.5	3.0	3.8	4.5	13.1
(40,45]	5.5	5.1	1.0	2.9	3.5	10.7
(45,50]	3.1	2.7	0.5	2.1	2.7	6.5
(50,55]	1.3	0.9	0.5	0.8	1.2	2.4
	**Female**					
	** Years to Next Visit at 5% **	** Years to Next Visit at 1% **	** Current Years to Next Visit **	** Number of Visits at 5% **	** Number of Visits at 1% **	** Current Number of Visits Until ** ** 50 mm **
[30,35]	7.8	6.5	3.0	5.9	52.5	13.2
(35,40]	4.1	1.8	1.0	5	51.4	10.7
(40,45]	2.2	0.1	1.0	3.2	47.4	8.3
(45,50]	3.1	0.7	0.5	1.3	5.9	4.2

Estimated optimal time to next visit by interval and total optimal number of visits considering that no more than 1% or 5% of the individuals would pass the threshold for surgery before that visit.

## Data Availability

The datasets presented in this article are not readily available due to privacy or ethical restrictions; however, they can be made available through a legal collaboration agreement. Further inquiries can be directed to the corresponding author. R scripts for mixed models and growth rate calculations have been shared through the following repository: https://github.com/tecnicUGCD/acaoaaagritsp, accessed on 20 June 2025.

## Supplementary Materials

The following supporting information can be downloaded at https://www.mdpi.com/article/10.3390/jcm14134720/s1: Table S1: Mean difference between CT and ultrasound before and after adjustment; Figure S1: Bland Alman tests with the corrected values, showing at 30, 60 and 90 days, showing very few datapoints exceeding the threshold (dashed red line) suggesting that there are no statistically significant differences between CT and ultrasound measurements. References [17,41] are cited in the supplementary materials.

## Abbreviations

The following abbreviations are used in this manuscript:

AAA	Abdominal Aortic Aneurysm
BMI	Body mass index
CIs	Confidence Intervals
CV	Other cardiovascular
CVE	Cerebrovascular event
COPD	Chronic obstructive pulmonary disease
DM	Diabetes mellitus
ESVS	European Society for Vascular Surgery
PAD	Peripheral artery disease
TABS	Triple-A Barcelona Study
