# Supplementary material for: A Comprehensive Analysis of the Abdominal Aortic Aneurysm Growth Rate in the Spanish Population
Source: J Clin Med. 2025 Jul 3;14(13):4720. doi: 10.3390/jcm14134720 (PMC12250419; doi:10.3390/jcm14134720)
Supplement: Supplementary file 1 [file jcm-14-04720-s001.zip › jcm-3707748-supplementary.pdf]

SUPPLEMENTARY MATERIAL

A comprehensive Analysis of Abdominal Aortic Aneurysm growth rate in the Spanish population

Growth rates of abdominal aortic aneurysm

Olga Peypoch MD MSc[1,2,3], Laura Calsina Juscafresa MD PhD[4], Antón Vega-Méndez[2], Bárbara Lobato-Delgado MSc[2], Joan Fité MD PhD[1,2], Begoña Soto MD PhD[1,2,], Luis Nieto MD[4], Mireia de la Rosa[2,] Ager Uribezubia[2], Jose Romero MD PhD[1,2], Emma Plana PhD[5], Manuel Miralles MD PhD[5,6,7], Albert Clarà MD PhD[4], Jaume Dilmé MD PhD[1,2,3,8], Jose Manuel Soria PhD[2,9], Mercedes Camacho PhD[2,8], Angel Martinez-Perez MSc[2,9], Maria Sabater-Lleal PhD[2,9,10]

METHODS

Sample inclusion and Data collection

From the moment of inclusion in the study, we performed control visits following recommendations from the European Society of Vascular Surgery unless additional medical advice for more frequent follow-up (every 3 years for diameters <40 mm, annually for diameters between 40-49 mm and every six months for diameters ≥ 50 until time of surgery for male, and every 3 years for diameters <40 mm, annually for diameters between 40-44 mm and every six months for diameters ≥ 45 until time of surgery for female) 13. Aortic diameter at the different follow-up visits was measured by ultrasound or CT-scan and all data were collected in the database.

Other relevant comorbidities were also obtained at inclusion through exploration of clinical records, and included presence/absence of dyslipidemia, hypertension, diabetes mellitus (DM), chronic obstructive pulmonary disease (COPD), cerebrovascular events (CVE), other cardiovascular (CV) events, peripheral artery disease (PAD), other aneurysms (including thoracic and visceral aortic aneurysms, iliac artery aneurysms, and popliteal artery aneurysms), or chronic renal failure. Finally, we documented current medication (antiaggregating drugs,

statins, anti-diabetic drugs, immunosuppressors, anti-inflammatory medication and anticoagulants), along with other relevant lifestyle and clinical information (smoking behavior, anthropometric measures) at each visit.

Additionally, cases that were scheduled for AAA surgery repair at the same hospitals were also included. For these patients, information from the diameter of the aorta was extracted from the clinical records of the patients, from the moment of inclusion in the registry (the moment when the aneurysm was detected) until the moment of the surgery (usually when the aneurysm exceeded 55 mm in men or 50 mm in women).

Finally, we included 40 individuals from Hospital Universitario La Fe biobank, with aortic diameter measures and clinical data at the time of surgery.

Ultrasound and CT-scan measures

Ultrasound measures of the diameter of infrarenal aortic artery was performed in all participants with the Philips EPIQ 7G ultrasound system. For all measurements, the maximum transverse diameter of the artery was reported in mm and was measured inner to inner by the same ultrasound-expert angiologist at each hospital.

All CT-scan images were obtained using Siemens Healthineers Somatom Force and Siemens Healthineers Somatom X.ceed scanners by a qualified radiologist, and diameters were verified by a vascular surgeon.

For the present study we excluded data from all visits that occurred after surgical interventions (both by endovascular aneurysm repair (EVAR) or open surgery).

To harmonize variability between CT-scan and ultrasound measurements, an adjustment was implemented to align ultrasound measurements with those obtained via CT-scan. Specifically, 2.21 mm was added to all ultrasound measurements. This adjustment factor was derived by analyzing the growth rates of aortic diameter (in mm/year) across four scenarios: consecutive

measurements performed exclusively with CT-scan, exclusively with ultrasound, transitioning from CT-scan to ultrasound, and transitioning from ultrasound to CT-scan. The growth rates were further adjusted to account for the dependence of growth dynamics on the baseline aortic diameter. Under the premise that the growth rate, corrected for diameter, should remain consistent across all measurement scenarios irrespective of imaging modality, a linear model was fitted to the data. This model demonstrated that an offset of 2.21 mm applied to ultrasound measurements was necessary to achieve the assumed uniform growth dynamics, confirming its validity as the adjustment factor.

In order to provide validation of the adjustment procedure we performed a Bland-Altman analysis comparing CT-scan and ultrasound -derived diameters both before and after applying the proposed correction.

Of note, because we rely on medical exams from the clinics and no paired CT and ultrasound measurements were available on the same date for the same individual, we identified measurement pairs from the same patient taken within 30, 60, or 90 days of each other, and where imaging modality (CT or ultrasound) changed. These pairs were used to estimate agreement between modalities. Table S1 shows the mean difference (bias) between CT and ultrasound decreased substantially after applying the correction cross all three time windows (30, 60, 90 days).

**Time Window**	**Mean Difference (Unadjusted)**	**Mean Difference (Adjusted)**	**p-value (t-test adjusted)**	**Proportion outside LoA**
30 days	+3.29 mm	+1.08 mm	0.50	1/17
60 days	+3.62 mm	+1.41 mm	0.08	2/45
90 days	+3.24 mm	+1.03 mm	0.07	3/79

Table S1: Mean difference between CT and ultrasound before and after adjustment.

Notably, after the adjustment, we observed that a) the mean difference approached 0 without significant deviation (e.g., $p = 0.07$ at 90 days), b) the limits of agreement remained stable and included 0, and c) no significant dependence of the difference on the average diameter was detected at 30 or 60 days.

Although a weak trend ($p = 0.0179$) emerged at 90 days, this likely reflects residual biological variation rather than measurement bias, since larger diameters are associated with higher growth rates, and CT scans are typically preferred as an imaging method in more advanced stages of the disease, when diameters are larger.

Taken together, this analysis confirms that the +2.21 mm correction effectively harmonizes the modalities, reducing systematic bias without introducing significant distortion. (Figure S1, Table S1).

Figure S1: Bland Alman tests with the corrected values, showing at 30, 60 and 90 days, showing very few datapoints exceeding the threshold (dashed red line) suggesting that there are no statistically significant differences between CT and ultrasound measurements.

A) Measures within 30 days

B) Measures within 60 days

C) Measures within 90 days

Calculation of the linear trend

To assess whether aneurysms followed a more exponential growth trend or if, oppositely, they tended towards stabilizing the growth rate, we calculated a measure of *growth trend* by dividing the area below the expected linear growth between the initial and final measurements with the area below the actual growth progression, and then multiplying by the *average growth rate* as calculated before, therefore emphasizing the growth value of subjects with an accelerating growth curve and diminishing the value of the subjects where the growth tended to stabilize.

$$Growth\ trend = \frac{(\Delta\emptyset \cdot \Delta t)/2}{\int (\emptyset(t) - \emptyset_{\min})dt}$$

Statistical modelling and selection of main factors associated to aortic diameter and growth rate

For the association models for aortic diameter, p-values were estimated using the R package *lmerTest*. Model-based marginal means (expected response variable for different combinations of predictor values) and adjusted predictions were estimated using the R package *ggeffects*. A mixed-effects model was previously fitted to account for both fixed and random effects, with aortic diameter as the outcome variable. Fixed effects included baseline aortic diameter, age, smoking status, and follow-up time, while random intercepts were introduced to account for within-subject correlation due to repeated measures.

We performed backward stepwise elimination of variables, including diseases, to obtain the most parsimonious model using the *buildmer* package. To test the goodness of fit of the most parsimonious model, we compared it with a baseline model that included only sex, age at study entry, and smoking as covariates, emulating the information currently being used in clinical practice to estimate aortic diameter.

For the growth rate models, we performed backward stepwise elimination of variables to obtain the most parsimonious model using the *buildmer* package. 95% Confidence Intervals (CIs) and p-values were computed using a Wald t-distribution approximation. To test the goodness of fit of the most parsimonious model, we compared all models' *REML Criterion* with a basic model that used only diameter at baseline, sex, age, and smoking as covariates, emulating the information currently being used in clinical practice to estimate aortic diameter.

Estimation of Time to Surgical Threshold

To estimate the time required to reach the predefined aortic diameter threshold for surgical consideration, we implemented the following approach:

For individuals who had already surpassed the threshold in one of their measurements, we estimated the time to reach the limit using linear interpolation between the last measurement below the threshold and the first measurement above it.

For individuals with limited data (<4 measurements), the time to reach the threshold was estimated using population-level mean growth rates of aortic diameter. This approach accounts for average growth trends while acknowledging the lack of individual trajectory data. For individuals with ≥4 measurements, we used individual linear regression models to predict the time to reach the threshold, leveraging the observed variability in their growth trajectories.

Estimation of extra visits saved and redirected to high risk group

We first estimated the number of visits that could be saved based on Figure 3, which estimates the time to surgery threshold considering that only 1% of the patients would surpass the surgery threshold. We then calculated the proportion of saved visits on total visits, considering the number of current visits now (430) in the area of influence of Hospital Sant Pau (400,000 inhabitants) and extrapolated to the total population with access to public healthcare in Spain in 2023 (47,755,450 individuals) (1), giving 38.03% of saved visits, which equals 19,523 potentially saved visits per year in Spain. Given that the prevalence of AAA in male smokers over 65 is 2.67% (2), if these freed resources were redirected to screen male smokers aged 60–65, this could allow the detection of approximately 521 new AAA cases per year in Spain.

(1) https://www.sanidad.gob.es/estadEstudios/estadisticas/sisInfSanSNS/tablasEstadisticas/InfAnualSNS2023/INFORME_ANUAL_2023.pdf

(2) Bravo-Merino L, González-Lozano N, Maroto-Salmón R, Meijide-Santos G, Suárez-Gil P, Fañanás-Mastral A. Validez de la ecografía abdominal en Atención Primaria

para detección de aneurisma de aorta abdominal en varones de entre 65 y 75 años. Aten Primaria. 2019 Jan;51(1):11–7.
